# Total pain and social suffering: marginalised Greenlanders' end-of-life in Denmark

**DOI:** 10.3389/fsoc.2023.1161021

**Published:** 2023-06-13

**Authors:** Vibeke Graven, Maja Bangsgaard Abrahams, Tina Pedersen

**Affiliations:** ^1^REHPA, The Danish Knowledge Centre for Rehabilitation and Palliative Care, Odense University Hospital, Nyborg, Denmark; ^2^Department of Clinical Research, University of Southern Denmark, Odense, Denmark

**Keywords:** total pain, social suffering, Greenlanders, marginalised Greenlanders, palliative care, stigma, othering, intersectionality

## Abstract

With a focus on socially marginalised Greenlanders in Denmark, this study explores the significance of the concept of social suffering for the concept of total pain. Greenland is a former Danish colony and Greenlanders retain the right to Danish citizenship with all the benefits of access to the resources of Denmark as any other Danish citizen. However, Greenlanders are overrepresented amongst the most socially disadvantaged in Denmark. They have a disproportionately high risk of early death, often undiagnosed and untreated. This study reports on research conducted with socially marginalised Greenlanders and some of the professionals who work with them. It interrogates the concept of total pain as developed by Cicely Saunders, the founder of modern palliative care. Saunders noted that pain at the end-of-life was not adequately explained by symptoms of a disease process because it was more like a situation that engulfed every aspect of the patient and those close to them; it included physical, psychological, spiritual, and social dimensions. We agree with other scholars that the social dimension of the total pain experience is underexplored. By drawing on the theoretical and methodological lens of intersectionality, our work with marginalised Greenlanders has enabled us to describe the multiple and intersecting social forces that create social suffering for this group. This leads us to conclude that social suffering is not entirely an individual experience but a product of social harm and disadvantage, poverty, inequality, and the various legacies of colonialism, which combine to place some citizens in a harmed condition. Our findings also draw us into a discussion with the concept of total pain and its neglect of the socially constructed nature of social suffering. We conclude by indicating ways in which the concept of total pain can be informed by a more thoroughgoing concept of social suffering. We conclude, with others, that there is a problem of inequity in the way that end-of-life care is currently distributed. Finally, we point to ways in which an understanding of social suffering can help to address the exclusion of some of the most vulnerable citizens from appropriate end-of-life care.

## Introduction

For more than 6 years, the first author has researched palliative care in Danish hospices where she found that paying attention to total pain often helps people to die peacefully and also to ‘live until they die'(Saunders, 2005) following the ideals of Cicely Saunders. However, patients were mainly middle-class white patients with cancer, a picture lately confirmed statistically (Adsersen et al., 2022). One, among several, wake up call that changed her focus from this cohort towards ‘socially marginalised Greenlanders'^1^ was the comment from a palliative care practitioner describing her visit to a Greenlandic woman with cancer she described as marginalised:

*As soon as I go through the door I can feel in my own reaction that all my prejudices are confirmed. You know, you enter a flat she has only had for a year, but totally smoky and yellow and you stink when you leave. Extremely dirty and sparsely furnished. She sleeps on the sofa surrounded by cigarettes and bottles. She is so lonely and somehow trapped there, she has a sparse network (…) I don't know if she was sober when we arrived, but we could only talk briefly and about simple issues. When I left I doubted whether she knew why we were there (…) working with the most socially marginalised people is complex and I experience it as personally frustrating because you would like to come with something that can help them*.

Apart from the problem of equity as to who receives appropriate care at the end-of-life, the narrative also points to the complexity of managing care at the end-of-life. Cicely Saunders, a nurse, social worker, and medical doctor, established St Christopher, the first modern hospice in 1967. She founded the hospice movement and has been a major inspiration for the development of the discipline and the culture of what today is internationally recognised as palliative care (Clark, 2018). Palliative care is defined by WHO as relief of serious health-related suffering, be it physical, psychological, social, or spiritual (Radbruch et al., 2020; World Health Organization, 2020). Saunders developed the concept of total pain inspired by what she learned from terminally ill cancer patients. Saunders explains that total pain ‘is a situation rather than an event' (Clark, 1999) and requires attention to the individual's physical, psychological, spiritual, and social pain. Clark (1999, 2018) points to how the concept challenged the dominant biomedical discourse and he gives an analogy of the sociological distinction between disease and illness. The concept of total pain focuses not only on somatic pain (disease) but also on the individual's narrative or subjective awareness (illness) (Clark, 1999; Wood, 2022). Even though ‘the social' is included in the definition of total pain, this dimension is the most underexplored (Rowley et al., 2021). In palliative care research, the social dimension is often conjoined as the ‘psychosocial' (Lormans et al., 2021) which arguably does not capture the social dimension fully.

We will take a wider and more structural approach to the social dimension as now recognised in the International Association of Hospice and Palliative Care (IAHPC) definition of palliative care with its focus on reducing barriers to palliative care (Radbruch et al., 2020). Thereby, we challenge the idea that social pain refers only to the experience of interpersonal connexions between the dying individual and the social world. The impact of social inequality and inequity is an increasingly important focus for palliative care (Stajduhar, 2020; Rowley et al., 2021; Richards, 2022; Sallnow et al., 2022). Inequity is defined as the unfair and avoidable differences in health status (World Health Organization, 2023). In line with what Richards describes as the ‘equity turn in palliative and end of life care research' (Richards, 2022), we will argue that the social dimension of total pain requires attention to suffering produced by the social world through inequality, marginalisation, injustice, powerlessness, and other structural forces.

Cicely Saunders developed total pain in response to a health system that had failed to manage the pain of dying people for whom nothing more could be done (Clark, 2018). However, in the same vein as Saunders (2005)' original provocation, there is a class of people excluded from palliative care. It is recognised that people at the end-of-life who also face social and structural inequities are less likely to access palliative care (Shulman et al., 2018; Davies et al., 2019, 2023; Stajduhar et al., 2019; Buck et al., 2020; Tobin et al., 2022). International research has identified some of the physical, psychological, social, and existential needs that characterise the end-of-life of socially marginalised people. Intersecting factors such as homelessness and substance use overlie issues such as fear of losing control, being a burden to others, dying alone, or remaining unidentified after death (Walsh, 2013; Ko et al., 2015; Rasmussen, 2017; Klop et al., 2018; Stajduhar et al., 2019; Graven, 2021). There are also concerns that professional services do not regard end-of-life care as a priority, lack the competence to manage complex pain alongside substance dependence, and the challenges of caring for socially isolated people who lack insight into their condition and have little or no family support (Klop et al., 2018; Stajduhar et al., 2019; Graven, 2021). Investigating the nature of ‘social suffering' provokes a new challenge for the concept of total pain.

Within the field of sociology, the connexion between suffering and social is theoretically well established. The concept of social suffering is used to describe how lived experiences of inequity and suppression can cause suffering. As such, the suffering is conditioned by the social that reaches beyond the subjective experience of suffering (Kleinman et al., 1997; Bourdieu, 1999; Wilkinson, 2005). Understanding social suffering thus involves directing the gaze towards the social conditions that cause suffering (Wilkinson, 2005; Wilkinson and Kleinman, 2016). As discussed by Wilkinson and Kleinman (2016): ‘The concern to understand human suffering in social terms brings critical debate both to the moral meaning of suffering and to the category of ‘the social” (Wilkinson and Kleinman, 2016). Including the perspective of social suffering in the approach to total pain invites an exploration of the social forces that affect total pain. In addition, a focus on social suffering is a springboard for questioning current understandings of total pain. With a focus on socially marginalised Greenlanders in Denmark, this study explores the significance of social suffering for total pain. In our study, socially marginalised Greenlanders are people whose pain must be explored in the shadow of a complex of social suffering understood as the sum of multiple structural factors including inequality, racism, and psychological and cultural trauma (Kleinman et al., 1997; Wilkinson, 2005; Subica and Link, 2022).

Greenland was colonised by Denmark in the 18th century (Barten and Mortensen, 2016) and not until 1953 was self-governance considered. In 2009, a new law established Greenland's autonomy within the Danish Commonwealth, recognising Greenlandic people as people in their own right, with the right of self-governance (Minority Rights Group, 2021; Statsministeriet, 2021). Greenland is the largest island in the world with a widely dispersed population. However, the indigenous Greenlandic population is not one homogeneous group; there are three main language groups, namely, the dominant West-Greenlandic (Kalaallisut), the minority East-Greenlandic, and Thule languages (Barten and Mortensen, 2016).

In 2021, 16,740 people born in Greenland lived in Denmark (Statistics Greenland, 2022). Greenlanders are Danish citizens with the same right of access to healthcare services as all Danes (Krusaa and Jacobsen, 2022). This includes a right to a translator if the *doctor* considers a translator necessary (Bekendtgørelse om Tolkebistand Efter Sundhedsloven, 2018). Greenlanders in Denmark have been discussed as an invisible and neglected group despite their status as Danish citizens because this very status means that their ethnicity is not registered in national registers which renders systematic assessment of their social and economic conditions difficult (Togeby, 2002; Nygaard-Christensen and Bjerge, 2021). However, surveys and mappings reveal an overrepresentation of Greenlandic people among the socially marginalised people in Denmark (Baviskar, 2015; Ahlmark et al., 2019). The average life expectancy of socially marginalised people, in general, is 19 years shorter than the average population (Strøbæk et al., 2017). It is well established that the experience of colonialism, and the long-term effects of being colonised, causes inequalities in the health status of indigenous people (Gracey and King, 2009; Subica and Link, 2022). Subica and Link point to cultural trauma as an additional causal factor in health disparities (Subica and Link, 2022). Several studies reveal that mainstream health services fail in meeting the needs of indigenous peoples and that indigenous people are often excluded and marginalised from mainstream health services (Harfield et al., 2018). This picture is also true for socially marginalised Greenlanders in Denmark who are overrepresented amongst those experiencing poverty and homelessness and those who are problematic users of alcohol and drugs (Ahlmark et al., 2019; Baviskar, 2015). Several Danish studies reveal structural barriers such as language, culture, ethnicity, and an inability to navigate Danish healthcare and welfare systems as factors in Greenlanders problematic access to welfare benefits (Togeby, 2002; Jakobsen and Larsen, 2014; Rådet for Socialt Udsatte, 2014; Baviskar, 2015; Social Boligstyrelsen, 2018; Ahlmark et al., 2019; Nygaard-Christensen and Bjerge, 2021; Krusaa and Jacobsen, 2022). A national survey of marginalised Greenlanders found that 50% of the respondents were homeless compared to 23% of the general population of people who are socially marginalised. The survey also found that 19% reported hunger due to poverty and almost half of the respondents had attempted suicide and that their contact with health and social care was primarily under acute circumstances (Ahlmark et al., 2019).

The aim of this study is to explore how ethnicity, socioeconomic status, and illness and death culture intersect and impact the end-of-life experiences of socially marginalised Greenlanders in Denmark. Palliative care research is beginning to recognise the cultural aspects of illness and death culture, and how suppression or ignorance of cultural identity can influence suffering at the end-of-life (Gunaratnam, 2013; Shabnam et al., 2021). These aspects are still underexplored and there is no research on these aspects for Greenlanders in Denmark. Recognising that total pain unfolds in the relationship between the dying individual and their social world, the study also aims to shed light on how the concept of total pain can be informed by understanding social suffering.

## Methodology and theoretical framework

Methodologically, the study is inspired by Layder's adaptive theory drawing on the idea that social reality is a complex, that cannot be captured by one explanatory principle, theoretical approach or methodology (Layder, 1998). The adaptive theory focuses on ties between the agency and social structures and the connexion between macro and micro levels of analysis in its approach to combining theory and empirical research. Adaptive theory transcends the gap between an inductive and deductive approach to research. The analysis is an ongoing process steered by theoretically informed ‘orienting concepts' that guide without being deterministic (Layder, 1998). Initially, our study used total pain and intersectionality as orienting concepts to focus our empirical work. However, following empirically informed insights into the social aspects of total pain, we expanded these to include ‘social suffering', ‘stigma', and ‘othering'. These orienting concepts are briefly introduced below.

### Intersectionality

Intersectionality provides a theoretical and methodological framework for analysing mechanisms of social suffering at the end-of-life. Using intersectionality enables an exploration of how lived experience intersects with the processes of power, structural vulnerabilities, and social identities (Collins, 1998; De los Reyes and Mulinari, 2005; Christensen and Jensen, 2012). As palliative care researchers increasingly recognise, the lens of intersectionality enables an exploration of the multiple levels of intersecting inequities that shape the end-of-life for marginalised people (Reimer-Kirkham et al., 2016; Rowley et al., 2021; Richards, 2022). To gain an understanding of social suffering as well as the role social structures play in marginalised Greenlanders lives, the methodology draws on Christensen and Jensen (2012) approach to intersectionality which employs life-storey narratives and the analysis of everyday-life (Christensen and Jensen, 2012).

### Total pain and social suffering

The concepts of total pain and social suffering work as orienting concepts for exploring how the social constitution of total pain is rooted in social situations and conditioned by cultural circumstances. As Wilkinson (2023) notes, ‘social worlds are inscribed upon the embodied experience of pain and there are many occasions where an individual's suffering should be taken as a manifestation of social structural oppression and/or collective experience of cultural trauma' (Wilkinson, 2023).

### Othering and stigma

The concepts of othering and stigma are also relevant to our research because they are contributors to social suffering through processes of differentiation. Jensen's definition of othering (Jensen, 2011) includes the notion of ‘intersectional othering', that othering is a discursive practise where powerful groups ascribe subordinate groups' problematic or inferior characteristics (Jensen, 2011). Othering is relevant to intersectionality since both concepts touch upon several different forms of social differentiation, thereby leading to intersectional othering (Jensen, 2011). Othering helps us to understand how power structures condition agency and to reflect on how historical symbolic meanings frame the possibilities for negotiating identity (Jensen, 2011). Therefore, the theoretical framework of intersectional othering allows us to explore how power structures and culture such as illness and death culture, socioeconomic status, and ethnicity intersect and influence total pain at the end-of-life for Greenlanders in Denmark.

To understand how socially marginalised Greenlanders experience stigma in everyday life, we draw on Link and Phelan's (Link and Phelan, 2001) theory of stigma. According to Link and Phelan, the four components that can produce stigma are: (1) labelling, (2) stereotyping, (3) separation, and (4) status loss and discrimination, when all four components are present in a power situation that permits them, stigma occurs (Link and Phelan, 2001). Discrimination can occur in two ways. First, it can exist as individual discrimination, where a labelled person can experience social inequalities in life circumstances and life chances. Second, it can occur as *structural* discrimination, where institutional practises directly or indirectly exclude labelled persons (Link and Phelan, 2001). As Link and Phelan observe, people are often socialised to absorb cultural stereotypes and therefore reproduce them (Link and Phelan, 2001). Moreover, it takes social, economic, and political power to stigmatise, and the stigma that people experience is shaped by the individual's power to resist stigma (Link and Phelan, 2001). The outcomes of stigma are different for different people, but stigma can lead to lower self-esteem, reduced access to social and healthcare services, and have an impact on life chances (Link and Phelan, 2001). Combined with intersectionality and othering, this stigma perspective allows us to grasp stigmatisation both on a structural and individual level. Hence, having a devalued status in the wider society can lead to stigmatisation enhancing social suffering at the end-of-life (Link and Phelan, 2001).

## Methods

Adopting an ethnographic approach enabled us to become immersed in the field and explore the social circumstances in which marginalised Greenlanders live and experience social suffering (Atkinson and Hammersley, 2007). This approach involved studying peoples' actions and accounts by participating in people's daily lives for an extended period of time (Atkinson and Hammersley, 2007).

Fieldwork took place in two cities in Denmark and involved participant observation and semi-structured interviews. VG conducted fieldwork over 18 months and was the responsible contact in all settings. She accessed the field in collaboration with local social services, NGOs, the Danish Cancer Society, and Greenlandic Houses^2^. Practitioners from these organisations acted as gatekeepers to the field. Participant observation focused on gaining knowledge of implicit beliefs, norms, and behaviours within the group (Atkinson and Hammersley, 2007). VG conducted participant observation in a social care-home (6 months), a social accommodation for people who need support from a social worker to manage everyday life (14 months), plus informal conversations with and observation of marginalised Greenlanders on the streets. This involved participating in and observing daily life such as meals, drinking, conversations, and professional care situations. For over a month, TP conducted participant observation by volunteering in two NGO drop-in centres that provide support for vulnerable peoples' basic needs plus social support. MBA conducted participant observation by volunteering over a month in a social café in a Greenlandic House. Practitioners who were gatekeepers to lay participants were asked the ‘surprise question' used in palliative care as a test for identifying patients nearing the last phase of life: ‘Would you be surprised if the person died within a year?' In this way, we were able to determine whether to include people in our study.

Eleven narrative interviews with lay participants and 13 semi-structured interviews with practitioners were conducted guided by bespoke interview guides for each group. The two participant groups consisted of (Saunders, 2005) lay Greenlanders who, in professional jargon, are labelled socially marginalised due to their socioeconomic status and social functioning; and (Adsersen et al., 2022) professionals and volunteers working in contexts in which Greenlanders with social problems live their everyday life (here called ‘practitioners'). Participants' names and professions are changed, and ages are collated in groups. Table 1 contains anonymised descriptions of participants in the study. Eleven of the participants have participated in narrative interviews whereas two are described based on field notes and fieldwork.

**Table 1:** Lay participants.

**Number**	**Name**	**Age**	**Years in DK and birth place**	**Accommodation type**	**Income**
1	Anna	50–55	40 years in DK, born in West Greenland	Social housing (apartment)^*^	Early retirement
2	Henrik	65–70	20 years in DK, born in Greenland	Social care home	Pension
3	Valborg	50–55	1 year in DK, born in West Greenland	Social housing	?
4	Dagmar	65–70	50 years in DK, born in West Greenland	Social housing (apartment)	?
5	Kim	55–60	13 years in DK, born in South-west Greenland	Social accommodation^**^	Early retirement
6	Malik	50–55	50 years in DK, born in South Greenland	Social accommodation	Early Retirement
7	Kristian	60–65	1 year in DK, born in West Greenland	Social housing (apartment)	Pension
8	Tilde	65–70	Born in South-west Greenland	Social care home	Pension
9	Knud	50–55	Born in East Greenland	Social care home	Early retirement
10	Anina	40–45	Born in Greenland	Homeless (dead)	No income
11	Maren	70–75	Born in Greenland	Social accommodation but factually homeless	?
12	Naja	65–70	Born in Greenland	Social housing (apartment)	Pension
13	Minik	65–70	20 years in DK. Born in Greenland.	Social housing (apartment)	Pension

^*^Social housing (apartment) could be a one-room dwelling. ^**^Social accommodation has social workers on site.

Table 2 contains anonymised descriptions of practitioners who have been interviewed.

**Table 2:** Practitioners.

**Area of speciality**	**Organisational context**	**Number (*n* = 29)**	**Method of interview**
Home care nurse	Municipality	4	Group interview face-to-face
Social nurse	Hospital based	1	Individual interview
Social worker	Drop-in centre	2	Group interview face-to-face
Social worker	Sheltered residence	1	Individual interview face-to-face
Social worker	Social care home	4	Group interview face-to-face
Social caretaker	Social accommodation	2	Group interview face-to-face
Social- and healthcare workers	Street and home level	2	Group interview face-to-face
Social workers and volunteers	Greenlandic house	12	Group interview face-to-face Individual interview face-to-face
Palliative care practitioner	Specialised palliative care	1	Individual interview face-to-face

### Accessing the field and ethical considerations

A key challenge is that of identifying and gaining access to seriously ill participants. The usual routes through established social and healthcare systems are problematic; as some seriously ill Greenlanders are undiagnosed or diagnosed so late that they are too ill to participate. Language is also a barrier because many Greenlanders are not fluent in Danish or prefer to use their mother tongue to better express themselves. Finally, amongst our participants, there was a significant issue of mental capacity both to consent to and to participate actively in the study. The factors here include heavy alcohol and drug use, as well as the impact of multiple traumas, all of which add to the problems of capacity but are also aspects of social suffering we seek to explore. Thus, the empirical objective to explore Greenlanders' experience of total pain is difficult to meet via the conventional institutional settings for end-of-life (Atkinson and Hammersley, 2007). Therefore, the research is conducted within the material world in which Greenlanders live their everyday life (Hastrup, 2003).

Being morally engaged in people's lives and being emotionally affected are necessary and inevitable aspects of being able to give voice to suffering (Kleinman et al., 1997; Bourdieu, 1999; Gunaratnam, 2012). However, in practise, it is an ongoing ethical challenge to navigate between the roles of observer and carer. Whilst writing this, VG communicates with Kristian who is desperate about his economic situation, his progressing cancer, and finding the help he needs. Helping him might not only help him as an individual but also gives an insight into the complexity of suffering that can bring public attention to essential components of suffering that otherwise would go unnoticed (Wilkinson, 2005). The potential blurring between research and personal involvement is an ever-present ethical risk that was navigated based on a pragmatic principle of trying to help people to get professional help.

Several of our participants were not fluent in Danish. In three interviews, we used a translator; however, in most cases, this was not an option as it was difficult to coordinate or find a trusted translator. Translations of various kinds were problematic, as Gunaratnam observes, and differences other than language such as class can affect communication and the interpretation of meaning significantly. In a less literal way, translating participants' pain experience through cultural grammar matched the linguistic challenge and we hope that our interpretation is veridical (Gunaratnam, 2003).

All participants gave their written or recorded verbal informed consent. The study was approved by the Research and Innovation Organisation, University of Southern Denmark. Journal no. 21/24661.

### Strategy of analysis

Data consist of field notes and transcribed interview data. All field notes were handwritten, and interviews were audio recorded and then transcribed (Brinkmann, 2013). The translation process from non-fluent Danish into comprehensible Danish and then into English has sometimes influenced the nuance of meaning or has lost some of the cultural framing which must be acknowledged as a consequence of this process. When data collection was finalised, all interview transcripts and field notes were coded in NVivo™ to provide provisional code labels associated with orienting concepts or provided empirically generated ideas (Layder, 1998). We then identified three main themes across data coded into the overarching theme of total pain. Afterward, we turned our focus to orienting concepts, which helped us to structure our analysis. Thus, the elaboration of the themes was a combination of inductive and deductive procedures that enabled us to elaborate on the concept of total pain (Gunaratnam, 2003).

### Analysis

The analysis has three sections. The first explores how socioeconomic status and ethnicity intersect in the lives of the participants as a complex web of differentiation associated with othering and stigmatisation. This provides a sociocultural backdrop that contextualises the conditions for social suffering at the end-of-life. Second, micro-level aspects of social suffering are explored by focusing on norms, symbolic meanings, and social interaction guiding the participants' interpretations and experiences of illness, dying, and death in their everyday life. Third, structural barriers to accessing and navigating within conventional institutional health and social care are explored in the light of individual and structural discrimination.

To connect our readers to our participants, each theme is introduced by a participant narrative (Wilkinson, 2005). The narratives also trouble the concept of social suffering. Living everyday life in a socially marginalised position involves resilience, and social suffering is not necessarily expressed as individual suffering. Finally, the narratives illustrate layers of complexity of social suffering, that social suffering intersects ethnicity, socioeconomic status, and illness and death culture.

### Othering and stigmatisation as part of everyday life

*Malik lived in social accommodation, with two rooms, a little kitchen, and a bathroom. In one room, there was a mattress with a dirty blanket. In the other, a few chairs and a little coffee table covered with beer cans and vodka bottles. He lit a candle on a beer can and said: ‘I always light a candle if I am here on my own. Then I don't feel so lonely'. He grew up in Greenland with his parents, but his father died when he was a little boy. ‘He died of heart failure. I ran after the ambulance and I cried and cried and cried'. His mother died of cancer when he was about 6 years old and he was sent to an orphanage in Denmark. Later, he went back to Greenland and lived with family.' I had a criminal youth; I also stole together with my family. I was the youngest and could get through the little window...we stole wine and beer and such things. I have been alcoholic since childhood; I was about six or seven'. ‘And now I have told myself that I should stop drinking, I also smoke cannabis. I am old, I am disabled, and I am also tired of being such a ‘sut' (sot) (…) Some days ago I could hardly walk and almost called the ambulance. But I tell myself that as long as I can walk I have to walk'. Malik had four children with two women but is not in touch with them. He hopes to make it to 60. ‘But if I die, so be it'. Malik said that when he was a child he had a near-to-death-experience and said: ‘It is strange, it is completely empty up there and a lot of small lights. They say I have to go back to earth again, and I came back to life. I felt that as a punishment.' VG: ‘What encourages you to get up in the morning?' Malik: ‘Beer.' He also told that he has his route that includes two drop-in-centres and a bench from which he sells The Big Issue. He would like to end his life in Greenland but he does not know where he would live: ‘But I have some friends I can live with'. He would like to get buried in Greenland. Half a year after the interview, Malik had become weaker. If he had the strength, he would still take his daily route. He lost his rollator but found a replacement somewhere. Malik's social worker said that: ‘When he drinks, he is in an extremely poor state. Then if he needs money or if there are some days he doesn't drink he seems to revitalise a little. (…) My intuition is that he knows it probably only goes in one direction.'*

In this section, we first identify characteristics of how social suffering manifests for our participants as compared to Danish socially marginalised people. Next, we describe a complex web of differentiation, intersecting ethnic and socioeconomic categories. This web operates interculturally, between Danish and Greenlandic cultures and within the group of Greenlanders.

Some lay participants bear the markers of social marginalisation such as alcohol and drug use, poverty, homelessness or supported accommodation, and an everyday life outwith conventional society. However, intersecting these markers are characteristics associated with being a *Greenlander*. Practitioners across the field describe the significant role of past traumas experienced by ‘their' Greenlanders that are more common and more extreme than in the equivalent Danish populations. One practitioner describes a complex pattern of trauma, abuse, poor social conditions, problematic social networks, and the impact of colonialism:

V: How do the traumas manifest themselves? G: Well, I think they do it in the way they live. That it's so massive, the abuse and their complete inability to maintain anything of what the rest of us consider normal: Being in control of their economy, having healthy close relationships. Many of them have close relationships but they are connected to [substance] abuse. Many Greenlandic citizens say that (...) you lose a network if you stop drinking. Many of them have lived a really hard life, also with physically hard labour. Also, they haven't had much money, also just to be homeless in Greenland! You get the feeling that there is a lot caused by their history in Greenland – that Denmark took over Greenland.

Malik's storey demonstrates this complex history and patterns of trauma, but this is not only an individual storey, many participants also describe experiences of bad deaths such as murder, suicide, shooting accidents, and early death by cancer and TB. A Greenlandic practitioner who describes her childhood in Greenland as the epitome of a socially marginalised background tells how she watched a family member beating her aunt to death and says: ‘I have stopped counting the number of people I used to know who have committed suicide'. Another Greenlandic practitioner describes how traumas experienced by some of ‘her citizens' cause death anxiety: ‘It's the background we carry with us. You become anxious of dying because you have experienced traumas and if somebody has died that way you think that you'll die in the same way.'

Practitioners describe how they need to show consideration for the traumas Greenlanders carry with them and avoid opening wounds, for example, by not talking about difficult things and as an explanation for limited participation in everyday-life activities. One practitioner described a traumatised social accommodation client: ‘Sometimes she gets these outbursts (…) she throws things around, hops and dances, shouts and screams. And then, when you talk to her it's all about somebody who has died, drowned or some family issues.' Fieldwork also gave an insight into how a history of trauma was used as an explanation for communication barriers and some people's reluctance to participate in social life. VG's field notes from a social accommodation state: ‘A woman has several times mentioned that she would like to talk to me. Today she says: ‘My head is Greenlandic today. I can't find the words in Danish' and clearly indicates that she wants to be left on her own'.

Some lay participants identify themselves with labels such as ‘alcoholic' and ‘cannabis-user'. Several practitioners comment on the extreme nature of these problems compared to similar Danish groups. As one practitioner comments: ‘It simply explodes – four bottles of vodka a day. It's suicide even though it takes time. It's so self-destructive. (…) We had somebody who was found with a blood alcohol level of 5 point something. It really is so extreme'.

These accounts show an aspect of suffering that cannot be reduced to individual tragedies. They also illustrate socially conditioned lived experiences of injustice linked with low socioeconomic status and the commonly described effects of colonialism (Kleinman et al., 1997; Subica and Link, 2022). Interrelated with these characteristics of social suffering are other processes of differentiation, which point to the extreme marginalisation of Greenlanders. Some of these processes are identified below.

The stigmatisation and intersectional othering of Greenlanders has racist connotations related to their ethnicity, what Goffman would describe as ‘tribal stigma' (Goffman, 1986) and compound by their low socioeconomic status. The process of othering is a reductionist discourse that others all Greenlandic participants to a few negative characteristics and by stereotype (Jensen, 2011). For example, othering by stereotype is captured in the phrase ‘grønlænderstiv' (Greenlander pissed). In a candid reflection on her own prejudices, a practitioner discloses a common Danish view of Greenlanders:

I have had prejudices, just like everyone else, like: ‘don't sit there and get grønlænderstiv'. And ‘Greenlanders are some wretched people, who sit on the benches and stumble around'. I might have said it myself once: ‘Yes, that's a typical Greenlander” and shrugged. But after I got to know some Greenlanders outside the abuse milieu, then I got a different storey which I'm able to use in my work. [How] they meet these prejudices even though they may be well settled with husband and kids, house and job and so on (…) you have to fight to get integrated and to be seen as equal.

This discourse of othering adds an ethnic stigma to suffering associated with living on the edge of society. The consequence of such stereotyping that link a specific behaviour to Greenlanders is an oversimplification of the internal variations within the whole group of ethnic Greenlandic people that lead to stigmatisation (Link and Phelan, 2001). How this discourse works as a stigmatisation process is indicated by the way all the practitioners with Greenlandic backgrounds describe similar experiences. One practitioner explains: ‘When I visit the shopping mall, some Danish people are suspicious of me. They seem to think I want to steal something (…) When I pass by, they turn their noses up, they expect me to smell of alcohol'. Another Greenlandic practitioner comments on how her experience of stigma clashes with the idea of Danish unity: ‘If we talk about Danish Commonwealth, there is no common at all because I still meet that gaze.' The practitioners from a Greenlandic background have the resilience to distance themselves from the stereotype and can reject the stigma by being explicit about its simplified view of Greenlanders in ways that our lay participants do not (Link and Phelan, 2001).

The othering and stigmatisation of Greenlanders are incorporated into various forms of differentiation. Whether an individual has the resources for resilience means the difference between distancing from or taking on a stereotype such as Greenlandic alcoholic. Several Greenlandic practitioners and translators describe how Greenlanders, who are socially integrated in Denmark and thus able to fight the stereotype, also tend to distance themselves from marginalised Greenlanders who appear to represent the stereotype. Malik's broken relationship with family members is one among several examples of the consequential exclusion: ‘My oldest son used to come and visit me sometimes, but I don't know where they live now. And my little brother, he says I'm a freak.' As such, differentiation that creates othering within the population of ethnic Greenlanders in Denmark leads to further exclusion of socially marginalised Greenlanders. How this perpetuation of the stigma works to exclude some Greenlanders was observed in a Greenlandic House to which access is only allowed if you are sober. MBA observed that the rule was displayed on posters and if somebody appeared drunk, they were reprimanded (FGH). Several lay participants avoided the exclusion by saying that they found the Greenlandic House ‘too posh' and did not like going there. Another differentiation is evident between Greenlandic and Danish socially marginalised people in which Greenlanders are regarded as the lowest in the hierarchy. In drop-in centres and social care-homes, Greenlanders are usually isolated from other groups, which can be seen both as a strategy for avoiding stigma and a consequence of it.

Another process of differentiation was identified within the group of our lay participants and reveals that the label marginalised Greenlanders does not refer to a homogeneous group. Several practitioners describe how Greenlanders take care of each other, but also emphasise that it ‘depends on whether they are from East or West Greenland” Some of the West Greenlandic participants articulate a stereotype of East Greenlanders as someone who ‘steals', are ‘cannibals', and are ‘lower' in the hierarchy and thus more or less excluded from the community. One example is Nora who is from East Greenland and lives in a social care-home. There are three other Greenlanders, and Nora is excluded from their community. As one practitioner commented: ‘They don't really want her (…) she is a cannibal, as they say. Tilde and Marie, they just can't get on with Nora. (…) They shout and yell: ‘fuck you'. Nora does that too'.

As described, various processes of stigma and othering influence social suffering associated with a marginalised status. This constrains the possibilities for agency due to a legacy of trauma plus the mechanisms of exclusion. Intersectional othering is a discourse related to ethnicity and social status defined by Danes that discriminates against and excludes Greenlanders from Danes and marginalised Greenlanders from well-integrated Greenlanders. This complex of stigmatisation and othering leaves the participants in an extremely marginalised position at the end of their lives. The next section focuses on everyday life with illness, dying, and death.

### Approaches to illness and death

*Anina came to Denmark to find her cousin. She had a 16-year-old pregnant daughter in Greenland. When VG first met her she was sleeping rough. She visited a drop-in centre to get a new coat because somebody stole the one she had left with her sleeping bag while she was having breakfast. For now, she wants to stay in [city] because she is having fun and has found a ‘boyfriend'. A few months later, VG joins her at a hospital appointment. She says that her boyfriend has beaten her up and she has been kicked out of the shelter for being too disruptive, and she is back on the street but in touch with the street-team and shelter. At the hospital, we had to wait for the scan. Anina was impatient and wanted to get back to the drop-in centre for lunch. However, with VG's persuasion, she stayed. Over the next couple of months, Anina moved in and out of shelters. About 3 months after the scan, Anina died from a brain tumour. A practitioner reported that in the last weeks, she drank as much as possible and died in a shelter surrounded by practitioners but without family*.

Anina was more focused on maintaining her network with other Greenlanders and keeping everyday routines such as meals in drop-in centres than she was on her health issues. However, her relative contentment with her life belies the conditions of suffering compounded by the social disadvantages that have narrowed her life chances to their current form. Social suffering affects the way illness and symptoms are approached and managed in everyday life (Kleinman, 1988). This part of the analysis explores norms, symbolic meanings, and social interaction guiding the participants' interpretations and experiences of illness, dying, and death in their everyday-life contexts.

### Coping with a life-threatening life

Several practitioners describe how Greenlanders cope with illness as a form of fatalism and a sign of their ‘toughness'. These attributes can be seen as a tendency to not respond to symptoms until they are severe and thus barriers to conventional treatment and pain relief. Maren, who spent most of her time on the streets, did not seek help when her health obviously deteriorated. A practitioner describes that when she was eventually hospitalised: ‘She had an abscess in her pelvic floor measuring 10 by 14 centimetres, severe infection and critically ill, and we do not know if she survives (…) the rest of us would have reacted earlier'. During her admission, she became increasingly introverted and stopped eating. About a week after hospitalisation, she died.

Another practitioner comments:

X has been hospitalised and was told that he is going to die if he does not take his heart medication, so we ask: ‘do you want to get this medication?', ‘No, no' (…) They are calmer in some way. Today I told a man: ‘Listen! You need to get the endoscopy, there is something wrong with your stomach', and he told me that he was feeling fine and that it's unnecessary. There would have been more panicking if it had been a Danish man.

Practitioners offer various for this fatalistic approach, as a cultural trait, as a result of stigma, or as a consequence of living a socially marginalised life:

My experience is that Greenlanders are very tough. They have to be really ill before they say something or ask for help. It's very often that they are walking around with something, which we don't detect until they are extremely ill (…) now I'm interpreting, but I think that they've been rejected and therefore they need to be very ill before they ask for help.

Several explanations point to a cultural identity that is less individualised than Danes and that, combined with social marginalisation, can foster an attitude of not being worth caring for. Anna who is in treatment for throat cancer describes this fatalistic approach in terms of a norm of managing-it-yourself:

VG: You like to manage it yourself? Anna: I do. For us Greenlanders you can say that we would rather manage it ourselves. VG: What does it take to accept help? Anna: I don't know. YES, if you cannot walk, then you need to get help. VG: But it's not something that you like? Anna: No, I don't like it. [asking for help] I don't, even though it's nice [to have help].

Malik describes a similar way of coping when he talks of ‘nearly' calling an ambulance because he was in acute pain. These behaviours might be seen as evidence of the ‘toughness' and ‘fatalism' that the practitioners describe. However, as one Greenlandic translator reports, being seen as sick is associated with a loss of social status (amongst Greenlanders) and people step back from the sick person. We have frequently observed lay participants going to enormous efforts to be seen as active members of their social group even when they are seriously ill. Anina does this even when diagnosed with brain cancer and Malik does this despite relying on a rollator. These acts of ‘toughness' can be seen as a way of preserving a social network which is a key source of belonging and identity.

### Social networks

Some of the participants are active within a social network. The data reveal a mixed picture of care from these networks when somebody gets ill. Anna is one among several examples of how illness can lead to social isolation and loneliness if it becomes impossible to continue routines and join the usual social network. VG asks Anna if her friends visited her after she began her chemotherapy:

Anna: No, no one calls me and asks if I'm feeling all right. VG: Do you wish they did? Anna: Yes, occasionally. Of course, some ask me, my family, that is it. VG: When you finish your chemotherapy, do you hope they'll contact you again? Anna: I don't care, it doesn't matter. Because when I got my chemotherapy, that was when I was in pain and needed them.

The social network does not necessarily disappear but the support it provides when somebody gets seriously ill mirrors the resources and role of the network and rarely provides the practical support the sick person may need. For a few days, Aida helps her uncle who lives in social accommodation. He is ill with TB and now has a broken shoulder. She says that she gives him his medication and cleans for him. She comments on his visitors. ‘They are just sitting there drinking, fucking and do nothing (…) Then they get grumpy but I tell them I need to clean and that they have to leave: ‘Can't you see that he is lying here in his bed'. A couple of days later, Aida gives up. The uncle can now leave his room. VG observes that he is irritated and tries to avoid Aida. Aida says: ‘I should help myself instead, I drink too much, my daughter is taking drugs and my son has started cutting himself'.

The most common support delivered by social networks was the provision of alcohol, cannabis, and food. A practitioner describes a woman:

She had her son who made some Greenlandic food, fish soup and such (…) also just to be there and see if she needed anything. But we also experienced that when she became very ill. There were not as many with her in the apartment. But they still contacted us because they were worried about her. Why we couldn't help. (…) So they really care about each other in a way I don't see with our other citizens with a different background.

Providing food is a practical measure but also of deeper significance linked to symbolic ideas of health. Traditional food is important for wellbeing and health, as Tilde says. She misses Greenlandic food and states that it is ‘for the body, we need that'. Material resources in the form of cannabis and alcohol are used as forms of self-medication. Kristian says that he believes cannabis might cure his cancer and that it helps with feeling sick after chemotherapy.

Practitioners comment that social network is sometimes a hindrance to compliance with medical treatment:

P1: They listen to each other. If you have schizophrenia, and need some medication and your friends say ‘you do not need that', then they believe it and rely more on their friends than on the health professional. It might also be that it is about health literacy. P2: It's so integrated in their culture to find support with each other.

This final comment alludes to an underlying solidarity between Greenlanders, as *Greenlanders* abide despite the exclusions. As described in a group conversation with Greenlandic practitioners and volunteers in a Greenlandic House: ‘We're cousins all of us Greenlanders. Almost everybody knows each other (…) It can be very far out because the network is so big. (…) Somebody might come here from Greenland to live with a friend's aunt, and they consider that family'. Yet, Nora and Anna are both examples of how different mechanisms of marginalisation exclude them from networking within the wider group of Greenlanders.

Even though the participants' social networks are characterised by the mechanisms of marginalisation, fieldwork reveals that funerals are a symbolic ritual that overcomes exclusion. The desire to show respect for the dead person and their family is a unifier across social groups of Greenlanders and activates a wide network. Honouring the dead and loved ones is crucial; as one Greenlandic practitioner says: ‘That is something we are brought up with. We have to accompany the dead to the grave no matter if we are family or not. Perhaps we know his aunt or whoever, and then everybody goes to the funeral for the last goodbye'. As such, death seems to dissolve the divide between socially marginalised and integrated Greenlanders. This stands in stark contrast to the funerals for socially marginalised persons with a Danish background where it is rare for more than a few people to attend.

### Dying, death, and spirituality

We found a complicated picture of approaches to dying and death. Tilde who lives in a social care-home describes how even though death is a frequent part of everyday life, there is a reluctance to discuss it:

Tilde: All you do is to go to one funeral after the other. Who's the next? When one is dead. They just die one after another (…). They die of drinking and TB, that's because they smoke cannabis, you know. Then they call it TB or cancer. There are so many I have known who have croaked. It's sad and unbearable to think about them. VG: Do you talk about it if somebody is seriously ill or dying. Tilde: No, you can't be bothered to hear that. If that is the problem, then I just leave, then, I don't know, then I just need a walk. The weather is fantastic right now because it's spring, then everything starts to blossom.

Tilde's abrupt change of subject is typical of responses experienced in other interviews and several practitioners also comment on Greenlanders' reluctance to talk about dying and death. A Greenlandic practitioner points to a possible cultural aspect of this in her reflection: ‘I think that [dying and death] is a touchy subject, but I also think that it's something which isn't a natural thing to talk about and can be a taboo. For example, if we talk about it, then death might come to us far too early. That is why you don't talk about death'.

For the researcher, it is not possible to know whether a ‘death taboo' is a deep cultural trait or a phenomenon that is observed in a marginalised group traumatised by frequent and sudden deaths. What is clear is that there are practical consequences, which complicate end-of-life care. Some participants have thought about their wishes for disposal but have not disclosed them to the practitioners involved in their care. An example is Kim who has children in Greenland. He says that he would like to be buried with his parents and brother in Greenland and that he wants to be cremated. VG: ‘Why is it important to be buried in Greenland – is it to be together again?' Kim: ‘Yes, with siblings and children.'

Several practitioners are influenced by the idea of openness about dying and death including end-of-life decision-making. However, some say that it is difficult to have such conversations with Greenlanders. Petra has heart failure and her 20-year-old daughter is her only relative. To support the daughter, a practitioner would like Petra to complete an expression of wishes for her death: ‘We cannot always talk about such things (forthcoming death). That is what I would like to do (…) how can we prepare your daughter to take all this responsibility?'

At this point, it is useful to reflect on the practitioner's frustration. As pointed out by Yang et al. (2007), stigma is embedded in the moral life of sufferers. The moral mode of experience is reflected in what matters most in everyday life and practical engagement. Lay participants are extremely marginalised, some of them are ruled by immediate needs given by the demands of substance dependency and thus forced to live life from 1 h to another. For lay participants, these conditions combined with trauma might play a role in marginalised Greenlanders' alienation from the conventions of planning for end-of-life. However, these observations do not mean that there is no awareness or planning for end-of-life as illustrated by Kim.

We have found that when asked about dying and death, participants are willing to talk about what is meaningful to them, their feelings about the ‘motherland', their relationship to nature, and the importance of kin and a shared heritage of spirituality. These responses give some insight into the various ways spirituality is integrated into the participants' view of life. However, the lack of recognition given to this aspect also reveals a hidden layer of othering of Greenlanders' cultural identity because it is neither understood nor recognised in some of the contexts where the participants live and die. A telling example is the symbolic meaning of nature. Viktor, who recently moved to a care-home located far from the fjord, where he used to go every day, says: ‘The water, nature, is where I connect to everything.' A Greenlandic practitioner also comments:

This is where hope comes in. Moreover, nature itself plays an important role in everyday-life for many Greenlanders. This is where you feel yourself, this is where you hear quietness, this is where you come into yourself. This is probably also where you find strength from yourself rather than resignation.

Nature, as a way of connecting to the world, echoes the life they used to live in Greenland. However, this is a tentative spirituality, a resource that has been overwritten by Danish culture and religion, as one Greenlandic practitioner states:

It's something from generations that has shaped it. Christianity has taken up so much space, but the truth I carry with me is my own (…) is somehow lying there deep in our hearts and can be opened (…) culture and our identity. It's our identity that we belong together and that we come from a place in which spirituality has lived for many years and has been forgotten because of Danish society that overtook our lives in that way.

For some lay participants, the adoption of Christianity, the religion of the dominant society, has brought solace and an avenue for expressing spirituality. For others, the connexion to traditional spiritual culture is fragmented but emerges in certain practises such as the tradition of name-giving. Although not a ubiquitous practise, it is important for understanding the wide approach to family and social networks, and for some, an underlying spirituality. A practitioner explains the spiritual meaning of names:'a person consists of three parts: a body, a soul and a name. It's the name that is a particular core part of the person, and that name can continue living in society by being given to a new-born person when somebody dies.' This spiritual approach to names was repressed by the Danish Christian mission (Nansen, 1894; Olsen, 2001).

Illness and death culture are adapted to meaning and resources accessible in everyday life. The participant's approaches to illness, dying, and death are interrelated to characteristics of living a life marginalised from conventional society and cultural aspects linked to Greenlandic traditions of family life and spirituality. This includes a reluctance of seeking help, talking about serious illness, dying, and death, plus limited help from social networks, which some practitioners describe as non-compliance behaviour. A spirituality rooted in traditional Greenlandic faith is indicated as an important part of some Greenlandic participants' cultural identity. It is vaguely articulated by lay participants and as a dimension that historically has been suppressed and neither known nor talked about by Danish practitioners, we term it a hidden layer of othering. In the next section, we will explore barriers to accessing and navigating the conventional health and social care system.

### Navigating health and social care systems

*Kristian had advanced cancer. He moved to Denmark 6 months before the first interview and previously worked as a civil servant in Greenland for 30 years. He did not identify himself as socially marginalised. He had a sister in Greenland and a brother in Denmark. He told me that when his doctors stopped treatment in Greenland, he thought: ‘I can't just wait here until I die'. He sold everything he had and moved to Denmark. A friend helped him to obtain his health insurance card, the GP referred him to the oncology unit, and he started outpatient treatment. The hospital referred him to a volunteer programme, which helped him to equip his home. Eight months after the first interview and until submission of this study, Kristian described an increasingly chaotic life. He struggled with financial matters and found it hard to understand who could help him. He stopped seeing the volunteer and his health deteriorated. The oncologist said that he would need chemotherapy for the rest of his life and that he needed to keep up his weight to continue in treatment. He commented: ‘I somehow hope they will [stop treatment]. I need a break. I'm going to discuss it with the oncologist today'. Kristian said that he was struggling with nausea and he had no money for food. ‘I've only got a little dry bread (…) Some days I cook for my friend and then we eat together. Often I don't get breakfast.' VG asked if he was able to get money for cannabis, he said he needed to prevent nausea. ‘I get some from friends and pay back when I've money. I spend too much money on cannabis (…) Somehow; I wish that I had never moved to Denmark. But I've nowhere to live in Greenland. However moving isn't realistic.' After having talked to the oncologist about his treatment, he sent a message: ‘I have talked to the doctor and he was busy so we didn't talk about the money issue.' Kristian did not want to go to a drop-in centre for food and financial advice: ‘I don't want to, it scares me, somehow.'*

Navigating the Danish healthcare and social care system is difficult for all lay participants like Kristian. The conventional institutions appear complex, with unfamiliar norms and procedures, guided by a logic that excludes lay participants. This section focuses on how this struggle with navigating the conventional institutional framework for health and social care can contribute to discrimination and stigma.

### Accessing the system

Kristian's trajectory is a social decline that parallels his progressive cancer. This is an example of how the Danish system is exclusionary and difficult to access for marginalised Greenlanders. This combined with a social network unable to be supportive in navigating the system risks leaving a dying person in poverty and despair. Kristian's financial problems mean that he has not paid his rent and cannot afford food. However, as Kristian reports it, his oncologist focused on his treatment and physical health leaving no time for talking about social problems. Neither has he had help with his social problems from the palliative care team. He describes the system as a ‘digital jungle'. A hospital nurse summarises the difficulties that marginalised Greenlanders face when they have to navigate the Danish system:

It think that we've systems that actually make high demands for the users to be able to access them and you've to be pretty efficient in society and have electronic access to invitations, knowing who your general practitioner is, having the ability to actually turn up to your appointment, just to be able to access the system, and having the capability to show up in an outpatient facility. I think that you make high demands to some of our weakest patient groups. They need to fit into a box, which they don't necessarily fit into because they for several reasons are so multi-burdened.

Some Greenlanders move to Denmark without a long-term plan. As Greenlanders are Danish citizens, they are not enrolled in an introductory programme as immigrants are. Therefore, they often, go under the radar as they do not know how to navigate a complex social system like that of Denmark. As one practitioner explains:

Sometimes Greenlanders think that it's fantastic to come to Denmark, and then they sleep on cousin Peter's couch the first year and earn a living by selling bottles. Then, all of a sudden, they have to get NemID and a medical card and get involved in Danish society, and that is really difficult.

Some of the practitioners mention simple barriers such as knowing which bus to use to go to the doctor and what side of the road to enter the bus. The challenges of accessing the Danish system are also caused by a clash of temporalities. Some lay participants, like Anina, operate according to a life lived from 1 h to another guided by what appears as meaningful in the present internal time (Schutz, 1967). However, this does not fit well with a system based on clock-time (Schutz, 1967) exact appointments, waiting time, etc. In addition, there can be a cultural aspect of lay participants' approach to time. A Greenlandic practitioner describes how when she first started her job in Denmark, she could not see the point of making appointments and organising her day in a diary. Although, she quickly realised that doing so was necessary. Like other lay participants, she refers to Greenlandic hunting culture as incorporated into Greenlanders' identity: ‘I think it comes from prehistoric times when the weather was the underlying basis for going out hunting. I think that it is still sitting there, instinctively.'

Practitioners across the field report that these different approaches to time often clash. One says: ‘For example if they receive a letter about an appointment at the hospital or something (…) without us knowing, well it is 105 percent certain that they don't get there in time.' A hospital-based practitioner describes that often patients discharge themselves and do not get the same support as other patients because they cannot fit in with the way the hospital is organised: ‘It's difficult to be allowed to [help them] because they can't function on a ward in which things are normal. I mean the rhythm of the day and the demands they have to face. They get so challenged because they feel that they're misread'. Also, individual discrimination is reported, as one practitioner from a social care-home describes: ‘Sometimes in relation to visiting the GP, we need to be very sharp and be their spokesperson, because sometimes we experience that they get dismissed: ‘It's because you drink', ‘it's because you smoke', ‘it's because you don't get decent food, you need to eat what they serve”.

### Language and understanding

Accessing the Danish system is further complicated by communication problems. Language difficulties are one of the most mentioned problems associated with communication between Danish practitioners and Greenlanders. Several participants find it difficult to communicate in Danish and this limits possibilities for understanding the nature of suffering. As a practitioner puts it: ‘It's a challenge with language. What I experience is that they pretend to understand in order to be polite'. Valborg describes her strategy when she sees the GP: ‘Even though I don't understand it, I just respond with ‘yes, yes' and ‘bye'. Several practitioners report that they have to speak a very ‘simple' language to Greenlanders to obtain some understanding. Greenlandic practitioners within the field point out that a translator is essential for mutual understanding, but it is given low priority. The frequent use of relatives as translators adds serious ethical challenges. A participant from Greenlandic House reports that when her mother was hospitalised, she was told to translate for her mother. For her, it was difficult because she wanted to be in the hospital as a daughter taking care of her mother and not as a translator. Danish-speaking practitioners often act as translators in conversations with GPs for example. A practitioner from social accommodation describes the complexity of communicating and that it requires knowledge about the person.

Actually, a lot of them are so bad functioning that they can't communicate with the GP. But often when the GP says something then we translate it, meaning that we repeat what was being said in a simpler way ensuring ‘do you understand that he asked if your pain is in the right or left side?' When it is spelled out it usually works. But for some of them we have the perception that even though we try to act as mediators, they don't understand a single thing of what is being said. In situations like that we try to supplement with our observations of the person. ‘We have noticed that she does this and this or complains about pain here'. It's not the optimal solution.

The importance of using a translator is pointed out by a Greenlandic practitioner: ‘When I talk to her, then she talks about all the things involving the problem. But when she has to explain it in Danish it only contours the problem she talks about, we don't get into the core of the problem.' The same practitioner illustrates the complexity of intersectionality in communication:

I know how to speak the language, but to understand their cognitive level – where are you placed. And then add alcohol or cannabis abuse. There are more layers, and I've to figure out, where are you, how much do I need to explain. Do I need to explain it to you as an adult, as a teenager or something simpler? Moreover, I have to dig up information about their health.

At the end-of-life, difficulties with communicating can leave the dying person alone in their pain. A nurse describes how with Maren it was difficult to know what she needed when she was in hospital. Her illness was advanced when she was finally hospitalised, and she did not speak Danish well and could not or did not express her needs. Another practitioner describes the difficulties of reading pain: ‘I remember the Greenlander we had. He was in such pain but he couldn't express it. Yes, he could express it by shouting but not through language.' Link and Phelan (Link and Phelan, 2001) describe that even in the absence of individual prejudice or discrimination, institutional practises can work to the disadvantage of minority groups and cause structural discrimination. For the lay participants, the difficulties of access and communication within the conventional health and social care system result in discrimination despite the principle of free and equal access.

## Discussion

Drawing on the experiences of our lay participants, we first discuss the nature of social suffering and how it relates to total pain. Second, we offer a more general discussion of how the concept of total pain can be theoretically informed by the concept of social suffering.

### Total pain for socially marginalised Greenlanders

Social suffering is an underexplored aspect of total pain. Our findings reveal the complexity of social suffering for an extremely marginalised group and point to how socioeconomic status and ethnicity work as intersecting factors which subordinated our lay participants by stigmatising and othering them (Link and Phelan, 2001; Jensen, 2011). For our lay participants, vulnerability to social suffering is the product of traumas, poverty, and exclusion that leaves them in a harmed condition, which shapes their end-of-life experience (Wilkinson and Kleinman, 2016). Those who have not already died an early death are likely to end their life undiagnosed, undertreated, and with limited access to appropriate care. Such an end-of-life trajectory affects total pain and we argue that due consideration needs to be given to the drivers of social suffering for marginalised Greenlanders at the end-of-life. Below, we will discuss how intersections of social factors work across micro, meso, and macro levels of society to cause social suffering and affect total pain.

On the micro level, social suffering is incorporated into lived experiences of illness, dying, and death (Kleinman, 1988). For Kristian, his economic challenges increasingly overshadowed his illness. However, our findings show that social suffering is often unarticulated; obscured by everyday-life challenges. Anina and Maren who lived on the street until shortly before their death were preoccupied with maintaining their everyday life rather than responding to their symptoms of illness.

To understand social suffering for lay participants, we need to understand how stigmatisation and othering work in their everyday life. As Yang et al. commented, attending to the moral experience of stigma, both for the stigmatised and the stigmatisers, reveals what is really at stake and what is threatened (Yang et al., 2007). The phrase grønlænderstiv shows not only the stigmatisation incorporated into the common language but also the power of ethnic Danes to categorise Greenlanders as subordinated others. Such is the power of stigma that those categorised often internalise the stigma themselves. Our findings only confirm the well-established facts that stigma threatens self-esteem, mental and physical health, plus access to healthcare systems (Link and Phelan, 2001; Yang et al., 2007).

As in other studies on socially marginalised groups' end-of-life experiences (Klop et al., 2018; Stajduhar et al., 2019; Graven, 2021), we also find institutional meso-level barriers preventing access to health and social care for lay participants. Our study, like other studies on indigenous groups, revealed that such barriers are intermeshed with communication problems related to language and cultural approaches to illness and death. In Denmark, there is a presumption that Greenlanders, as Danish citizens, will know how to navigate the health and social systems.

Social forces on a larger or macro scale are also in operation. The long shadow of colonialism and modernisation should be acknowledged as contributing to stigmatisation, othering, and exclusion of Greenlanders. However, some commentators have cautioned against the identification of Greenlanders as a specific target group. Nygaard-Christensen and Bjerge are critical of over-emphasising culture and cultural differences as contributing factors in relation to problems experienced by Greenlandic people (Nygaard-Christensen and Bjerge, 2021). They point to the risk of alienating Greenlandic people even further and overshadowing more diverse representations that foreground the experiences of Greenlandic minorities. However, there is also a risk of not giving due consideration to these factors (Jenkins, 1997). We are not claiming a kind of Greenlander exceptionalism but rather seek to shine a spotlight on a highly marginalised group to unpack the concept of social suffering and how it illustrates the intersections of social factors that create such harmed states.

Our participants' (proudly) identify themselves as Greenlanders though their life storeys can be read as lived examples of a cultural/historic struggle with Danish culture. The focus on Greenlanders is important because the character of social problems for Greenlanders is more extreme than for other socially marginalised Danes (alcohol consumption and experienced trauma). Among Danish citizens' attributed low status and power, Greenlanders rank lowest, with all the implications for life chances that entail (Link and Phelan, 2001). Subica and Link's causal model of health disparities identifies cultural trauma as a relevant causal factor that can address the social suffering we have identified (Subica and Link, 2022). They define cultural trauma: ‘as an overwhelming and often ongoing physical or psychological assault or stressor perpetuated by an oppressive dominant group on the culture of a group of people sharing a specific shared identity/affiliation (e.g., race/ethnicity, nationality, religion)' (Subica and Link, 2022). Cultural trauma can be intergenerational and affect health disparities via stigmatisation of affected groups and decreased access to flexible resources. This pattern reflects the characteristics of lay participants in our study, illustrative of what Duran et al. describe as a ‘soul wound' (Duran et al., 1998; Subica and Link, 2022). Soul wound refers to a'deep psychological injury that directly compromises people's sense of wellbeing, safety, self-regard, and coping (…) manifesting in problems such as depression, anxiety, shared posttraumatic stress, and substance use' (Subica and Link, 2022). It is no coincidence that the age range of our lay participants places them in a cohort of Greenlanders born in 1950s onwards for which there is statistical evidence of cultural trauma. The rate of suicide, alcohol use, and domestic violence increased massively in the 1950s, coincident with the decolonisation and modernisation of Greenland (Larsen et al., 2019). This picture is consistent with the personal storeys of our participants who describe suicide, violence, and alcohol use as some of their earliest experiences. Our point here is that the cultural heritage of our lay participants is relevant for understanding the psychological traumas that are known to affect symptom burden at the end-of-life (Feldman and Periyakoil, 2006; Ganzel, 2016).

Subica and Link also point to the degradation of cultural resources in the form of cultural modes of being including customs values and spiritual beliefs (Subica and Link, 2022). Our study also points to the suppression and disregard of traditional spirituality that went along with colonialism and missionary activity that were part of the Danification of Greenlanders in the post-1950s modernisation. Most of our lay participants experiencing such traumas exemplify the complexity of individual and intergenerational cultural aspects that affect end-of-life for marginalised Greenlanders. Recognising the cultural trauma of indigenous groups as a factor in social suffering also brings insights into the nature of total pain for marginalised Greenlanders. Physical pain, social isolation, plus psychological and spiritual distress cannot solely be read as the person's symptoms given by a life-threatening illness; for the person with a cultural wound, total pain has to be understood in the light of a person's harmed cultural identity with the consequences it might have for living with life-threatening illness.

Drawing on findings from our study, we have shown that social suffering is an important aspect of total pain. This takes us to the question: How can total pain be informed by the concept of social suffering?

### The concept of total pain

As a starting point, it is helpful to come back to the origin of total pain. Saunders developed the concept as a way of thinking through the complexity of pain and as a strategy for managing pain. As Clark comments, Saunders recognised that: ‘there may be several layers which have to be understood to have a full grasp of the problem of pain in the terminally ill' noting also that pain is indivisible from both body and the wider personality (Clark, 2018). Even though it has been criticised for its slipperiness, it has been an inspirational tool for palliative care (Clark, 2018; Krawczyk et al., 2018; Wood, 2022).

Inviting the concept of social suffering into the discussion of total pain does not deliver a full theoretical framework for total pain; it nevertheless makes an important contribution to filling the theory gap. As we have discussed, social suffering shapes the nature of suffering that comes with dying. To grasp the core problem of socially produced suffering, it is required to understand how social forces of oppression such as cultural trauma and poverty work. This is a dimension mostly ignored in the literature that deals with the social aspects of total pain (Lormans et al., 2021). Aligning the social with the psychosocial definition emphasises individual experiences at the expense of the social understood as intersecting social forces. Social suffering is by definition produced by the social and it cannot be reduced to something within the person. This approach to the social has consequences for practise and here we point to just two implications. The first concerns the increasingly recognised problem that palliative care is an unfairly distributed privilege. While it is of course right that palliative care continues to try to understand the individual experience of pain, it is strategically problematic for palliative care to be blind to the social forces that determine who receives palliative care (Stajduhar, 2020; Rowley et al., 2021). Studying marginalised Greenlanders' social conditions has revealed inequity that calls for structural changes. We can only agree with Stajduhar that these inequities challenge the very foundations of hospice care because ‘it is failing to care for the people who suffer most at the end of life' (Stajduhar, 2020).

The second implication for practise is more abstract but concerns the use of the social lens to try and understand what is at stake when a socially marginalised person, for example, presents to palliative care. Attempting to understand the other in terms of the social forces that have shaped their experience, like Malik, who has been exposed to Danification, trauma, and alcoholism, requires knowledge and recognition. We began this study with a doctor's account of visiting a terminally ill Greenlandic woman at home: ‘*As soon as I go through the door I can feel in my own reaction that all my prejudices are confirmed* ‘—this comment can be interpreted as an instance of othering. When othering occurs, we already restrict the possibilities of understanding. There is a challenge here that must be addressed: the necessity but near impossibility of understanding the full meaning of pain. When Gunaratnam brings the concept of social suffering into conversation with total pain she points to how the very awareness of the limitations of understanding the other can actually be an opportunity for developing new ways of bridging and communication (Gunaratnam, 2012). Bringing social suffering into the ontology of total pain is perhaps a small step towards bridging the gap in understanding the complex suffering at the end-of-life experiences of socially marginalised people.

## Conclusion

This study is the product of a long journey towards a still incomplete understanding of social suffering for people labelled as socially marginalised Greenlanders. As white middle-class Danish-speaking researchers, we have been confronted by the limit of what can be known about the other. An additional challenge has been that our participants are silent on the issue of social suffering, for they rarely complain about their situation not even when confronted by life-threatening illness. Although our participants say little, it is their situation that speaks volumes, as Greenlanders, as Danish citizens, and as socially precarious people, they are situated at the intersection of multiple forces.

Significant in our findings are the following: (1) The history of colonialisation and modernisation that has inflicted a cultural wound that cannot be ignored as a major contributor to social suffering. The long shadow of colonialism has, to some degree, alienated Greenlanders from language, spirituality, and cultural identity; (2) Social cultures of care and community have, for our participants, become adapted to a culture of everyday life, frequently dictated by the demands of maintaining fragile networks and marginalised from conventional society; (3) The paradox that as Danish citizens they have rightful access to high-quality health and social care, yet the organisational logic of these systems makes them difficult to access without the appropriate literacy to navigate the system; and (4) The illness and death culture of our participants appear to be tough and stoical, but we need more knowledge to understand the intersecting factors constructing this facade.

Our work with marginalised Greenlanders, many of whom died early, often undiagnosed and untreated, has also provided a platform from which to reflect on the nature of suffering and the topic of this volume, total pain. One important observation is that total pain, as currently theorised, says little about the social construction of suffering. As we have noted, some of our participants are silent about their suffering, but pain and suffering are not solely individual experiences. Understanding social suffering requires an awareness of the multiple social forces that intersect to create a harmed condition, which in the case of our participants so frequently excludes them from attention to their needs at the end-of-life.

## Data availability statement

The datasets presented in this article are not readily available because the data are confidential to the researchers as consent is not in place for data sharing. Requests to access the datasets should be directed to vibeke.graven@rsyd.dk.

## Ethics statement

The studies involving human participants were reviewed and approved by the Danish Data Protection Agency, Region of Southern Denmark Journal no.: 21/24661. Written informed consent or oral recorded informed consent was obtained for participation in this study in accordance with the national legislation and the institutional requirements.

## Author contributions

VG, TP, and MA conceived, designed the analysis, collected the data, and developed analysis tools. VG performed the analysis commentated by TP and MA. VG and TP wrote the manuscript. All authors contributed to the article and approved the submitted version.

## Footnotes

^1^We use ‘scare' quotation marks on the first use of this problematic phrase, which is drawn from the policy and practice literature and we are aware that the phrase may contribute to othering. In referring to our participants, we use the phrase ‘lay participants'.

^2^Greenland's department for education and social affairs support four Greenlandic Houses in Denmark, providing cultural contact between Greenlanders and Danes. Social, legal, and educational counselling are provided along with social cafes targeted at vulnerable groups.
